# Pathways to performance in undergraduate medical students: role of conscientiousness and the perceived educational environment

**DOI:** 10.1007/s10459-021-10059-6

**Published:** 2021-07-22

**Authors:** S. Schrempft, G. Piumatti, M. W. Gerbase, A. Baroffio

**Affiliations:** 1 Unit of Development and Research in Medical Education (UDREM), Faculty of Medicine, University of Geneva, Rue Michel Servet 1, 1211 Geneva 4, Switzerland; 2 Unit of Population Epidemiology, Division of Primary Care, Geneva University Hospitals, Geneva, Switzerland; 3 Institute of Public Health, Faculty of BioMedicine, Università della Svizzera italiana, Lugano, Switzerland

**Keywords:** Academic performance, Conscientiousness, Learning approaches, Medical school, Objective structured clinical exam, Perceived educational environment, Undergraduate medical students

## Abstract

This study examined conscientiousness and the perceived educational environment as independent and interactive predictors of medical students’ performance within Biggs’ theoretical model of learning. Conscientiousness, the perceived educational environment, and learning approaches were assessed at the beginning of the third year in 268 medical students at the University of Geneva, Switzerland. Performance was examined at the end of the third year via a computer-based assessment (CBA) and the Objective Structured Clinical Examination (OSCE). Path analysis was used to test the proposed model, whereby conscientiousness and the perceived educational environment predicted performance directly and indirectly via students’ learning approaches. A second model included interaction effects. The proposed model provided the best fit and explained 45% of the variance in CBA performance, and 23% of the variance in OSCE performance. Conscientiousness positively predicted CBA performance directly (β = 0.19, *p* < 0.001) and indirectly via a deep learning approach (β = 0.05, *p* = 0.012). The perceived educational environment positively predicted CBA performance indirectly only (β = 0.02, *p* = 0.011). Neither conscientiousness nor the perceived educational environment predicted OSCE performance. Model 2 had acceptable, but less optimal fit. In this model, there was a significant cross-over interaction effect (β = 0.16, *p* < 0.01): conscientiousness positively predicted OSCE performance when perceptions of the educational environment were the most positive, but negatively predicted performance when perceptions were the least positive. The findings suggest that both conscientiousness and perceptions of the educational environment predict CBA performance. Research should further examine interactions between personality traits and the medical school environment to inform strategies aimed at improving OSCE performance.

## Introduction

There has been a long-standing research focus on factors that predict academic performance during medical school (Ferguson et al., 2002), and for good reason. Performance in the early years of medical school predicts residency performance (Lee & Vermillion, 2018), performance on the Medical Licensing Exam (Rubright et al., 2019), likelihood of student dropout (O’Neill et al., 2011), and risk for professional misconduct (Papadakis et al., 2005; Yates & James, 2010). Identifying pathways through which factors predict medical student performance remains a priority in medical education research.

Biggs’ 3P (Presage-Process-Product) model of learning (Biggs, 1993a) is an influential model in the field of higher education, and provides a framework for examining pathways to student performance. The model posits that students adopt qualitatively different approaches to learning (process factors), which vary as a function of intra-personal presage factors (including prior ability, sociodemographic characteristics, and personality), and contextual presage factors (including the teaching and learning environment). Presage factors may influence performance outcomes (product factors) directly, and/or indirectly via process factors. Research based on the 3P model has typically identified two approaches to learning: (1) A deep learning approach, whereby students conceptually analyze and draw meaning from the learning materials, often driven by an intrinsic interest in the topic. (2) A surface approach, whereby students use strategies that will meet the minimum requirements needed to complete the task, often motivated by extrinsic factors such as the need to pass a test (Biggs, 1993b; Biggs et al., 2001). In addition to direct and indirect predictive effects proceeding from presage to process to product, the model proposes that there are feedback relationships between each stage: products affect future process and presage variables, and process variables can affect some presage variables.

The original 3P model has been expanded to include students’ perceptions of their educational environment, proposing that the medical school environment influences students’ perceptions, which in turn influence their learning approaches, and ultimately their performance (Price, 2014; Price & Richardson, 2004). Indeed, students with more positive perceptions of the educational environment (including their perception of workload, relevance of teaching to professional practice, and social support systems within the school) are more intrinsically motivated (Muller & Louw, 2004), adopt deeper approaches to learning (Baeten et al., 2010), and perform better in medical school (Wayne et al., 2013) than students with less favourable perceptions.

Research examining predictors of student learning approaches and performance outcomes within the 3P framework has largely developed along two strands: the first focuses on student characteristics such as personality (Chamorro-Premuzic & Furnham, 2008; Diseth, 2003; Duff et al., 2004; Komarraju et al., 2011); the second on student perceptions of the educational environment (Entwistle, 2009; Ramsden, 1991). This division may reflect the idea that student characteristics and contextual factors are unique influences on approaches to learning (Biggs 1987; Newble & Entwistle 1986; Sadlo & Richardson, 2003). However, for a comprehensive understanding of pathways leading to performance, researchers should incorporate both student characteristics and aspects of the learning environment into their analysis models.

Just two studies have incorporated both personality and the perceived educational environment as presage factors in tests of the 3P model of learning (Ginns et al., 2014, 2018). Using data from Australian high-school students, these studies found that both the perceived educational environment (perceived teaching support) and personality (conscientiousness) had direct effects on product outcomes (class participation, homework completion, and educational aspirations), as well as indirect effects on the same product outcomes via elaboration and memorization. There are no such studies in the context of medical student performance, and no studies have examined potential interactions between these factors. Both personality and the perceived educational environment have been identified as key predictors of medical student performance (Doherty & Nugent 2011; Wayne et al., 2013), and it is important to understand factors that may mediate this association. Moreover, the interaction of individual- and context-level factors likely reflects the true complexity of student performance (Gruppen & Stansfield, 2016) and should be formally tested within models of learning.

Typically, research within the 3P framework identifies personality (alongside sociodemographic characteristics, such as age and gender) as a ‘hard’ presage factor, not easily changed by teaching (Biggs et al, 2001). However, advances in personality theory contrast with this view, drawing on evidence that trait expression is context-dependent, and traits can change over time (Dingemanse et al., 2010; Ferguson et al., 2011; Ferguson & Lievens, 2017). For example, there is some evidence that high conscientiousness enhances performance during the pre-clinical years of medical studies (when the learning context is primarily classroom-based, and knowledge is assessed by exams), but not during the clinical years (when students have to apply their knowledge to diagnose and treat patients, requiring greater flexibility of thought) (Ferguson et al., 2014). It is conceivable that the effects of personality on performance will vary according to the perceived educational environment. According to Trait Activation Theory, the behavioral expression of traits depends on the presence of trait-relevant contextual cues (Tett et al., 2013, 2021). Therefore, students may be most likely to perform well when their personality traits facilitate performance, such as when conscientiousness is high, and when they perceive their learning environment to be supportive.

In addition to the gap outlined above, most of the aforementioned studies on the 3P model only examined one-way learning interactions (from presage, to process, to product), without considering reciprocal relationships between presage and process factors, and therefore provided a less robust test of the proposed model (Anderson & Gerbing, 1988; Platt, 1964). Recent cross-sectional and longitudinal studies indicate that student learning approaches can act as presage factors predicting students’ experiences of the teaching–learning environment, as well as process factors predicting performance (Fryer & Ginns, 2018; Karagiannopoulou & Milienos, 2015). There is also evidence that personality predicts the perceived educational environment (Gruppen & Stansfield, 2016; Nijhuis et al., 2007), and it is important to consider this pathway to performance.

The main aim of this study was to use Biggs’ 3P model as a framework for examining pathways to performance in the context of medical education. Both conscientiousness and student perceptions of the educational environment were incorporated into the tested models, permitting an understanding of their relative and interactive contributions to student performance. The following three questions were addressed:

1. Do conscientiousness and the perceived educational environment have direct effects on performance, as well as indirect effects on performance via learning approaches (the main pathways highlighted in the 3P model)?
2. Are there interactive effects of conscientiousness and the perceived educational environment on learning approaches and performance?
3. Do conscientiousness and learning approaches have direct effects on performance, as well as indirect effects on performance via the perceived educational environment?

It was hypothesized that the findings would be in line with the main pathways highlighted in Biggs’ 3P model of learning, corroborating previous research on high-school students (Ginns et al., 2014, 2018). Specifically, it was hypothesized that conscientiousness and the perceived environment would have direct effects on performance, as well as indirect effects on performance via learning approaches. It was also hypothesized that there would be an interactive effect of conscientiousness and the perceived environment on student performance, in line with Trait Activation Theory (Tett et al., 2013; 2021) and findings that the impact of conscientiousness on medical student performance is context-dependent (Ferguson et al., 2014). Specifically, it was hypothesized that performance would be highest among highly conscientious students who perceived their educational environment to be supportive. Finally, based on the evidence available (Fryer & Ginns 2018; Gruppen & Stansfield 2016; Karagiannopoulou & Milienos 2015; Nijhuis et al., 2007), it was hypothesized that both conscientiousness and approaches to learning would have direct effects on performance, as well as indirect effects on performance via perceptions of the educational environment.

## Method

### Participants

Participants were 268 third-year medical students (108 males and 160 females; mean age 21 years (SD = 1.88, range = 17–38 years)) enrolled in the academic selection years 2013–2014, 2014–2015, and 2015–2016 at the University of Geneva, Switzerland. The sample was part of a larger research project at the University of Geneva designed to follow a cohort of medical students throughout the curriculum. Students participated on a voluntary basis, and gave their informed consent before completing the questionnaires. The Chair of the Cantonal Commission for Ethical Research (CCER) designated the study as exempted from formal review, in accordance with the Swiss law.

### Measures

#### Conscientiousness

Conscientiousness was assessed using the French version of the NEO-Five Factor Inventory (NEO-FFI), a self-report questionnaire designed to capture the ‘Big Five’ personality traits (Costa & McCrae, 1992; Rolland et al., 1998). Twelve items measure conscientiousness, and each item is scored on a 5-point scale (1 = strongly disagree, 5 = strongly agree). Higher scores reflect higher levels of conscientiousness.

#### Perceived educational environment

Student perceptions of the educational environment were captured using a French version of The Dundee Ready Educational Environment Measure (DREEM; (Roff et al., 1997)). The French version used for this study was a professional translation from the validated English version at the Ottawa University (courtesy of Timothy Willett). This is a 50-item self-report questionnaire, consisting of five subscales: the perceived learning environment (12 items; e.g., “the teaching is student centered”), the perceived teaching environment (11 items; e.g., “the teachers are good at providing feedback to the students”), the perceived atmosphere (12 items; e.g., “the atmosphere motivates me as a learner”), academic self-perceptions (8 items; e.g., “I feel I am being well prepared for my profession”), and social self-perceptions (7 items; e.g., “there is a good support system for students who get stressed”). The total score is the sum of all items, with higher scores reflecting more positive perceptions of the educational environment. The DREEM has been used extensively in the context of medical education (Colbert-Getz et al., 2014), and is reliable across countries, cultures, and settings (Soemantri et al., 2010).

#### Learning approaches

Student learning approaches were measured using a French version of the Revised Two-Factor Study Process Questionnaire (R2-SPQ) (Biggs et al., 2001). A French version of the questionnaire was not available at the time of the study, therefore the items were translated into French and back-translated into English by two independent reviewers. The questionnaire comprises 20 items, each scored on a 5-point scale (1 = this item is never or only rarely true of me, 5 = this item is always or almost always true of me). Ten items assess a deep learning approach (e.g., “I find most new topics interesting and often spend extra time trying to obtain more information about them”), and 10 items assess a surface learning approach (e.g., “I find I can get by in most assessments by memorizing key sections rather than trying to understand them”).

#### Performance

The medical school curriculum at the University of Geneva consists of a 3-year Bachelor program and a 3-year Master program. In the third year of the bachelor program, there are summative assessments at the end of each module (twice per year). Module 3 (Introduction to the nervous system; Neurosciences) is assessed at the end of the first semester, and module 4 (Defence and immunity; Infections) is assessed at the end of the second semester. Assessments include computer-based written exams (CBAs), and Objective Structured Clinical Exams (OSCEs). The CBAs cover the topics taught during the module, using vignettes and multiple-choice questions. The OSCE is an oral exam assessing clinical skills taught in the second and third years of the Bachelor. Students pass three stations with standardized simulated patients.

Student performance on the CBA of module 4, and their performance on the OSCE were the main outcome variables in this study. These outcomes were assessed at the end of the second semester, after the students had completed the self-report questionnaires (on conscientiousness, the perceived educational environment, and learning approaches). Module 3 CBA performance was assessed before the self-report questionnaires; therefore, this was included as a covariate rather than an outcome variable.

#### Covariates

The 3P model identifies sociodemographic characteristics (including age, gender, and parental education), and previous performance, as additional presage factors relevant to performance outcomes. Indeed, these sociodemographic characteristics have been associated with student learning approaches and performance (Richardson, 2006; Richardson et al., 2012), and previous performance is a strong predictor of future performance (Stegers‐Jager et al., 2015). These factors were therefore included as covariates. Parental education level (highest obtained level of mother and father) was categorized as lower (no qualifications, high-school education, or vocational education), or higher (university education). Performance on the CBA at the end of the first semester (assessing module 3) was used as a recent measure of prior performance. Repeater status (whether or not the student had re-taken the first year of medical school) was also included as a covariate.

### Statistical analyses

Path analysis was used to examine presage factors as predictors of product factors in 3 proposed models. Model 1 examined conscientiousness and the perceived educational environment as presage factors predicting performance directly and indirectly via student learning approaches. Model 2 added an interaction term (conscientiousness X perceived environment) to model 1 to test for potential moderating mechanisms. Model 3 examined conscientiousness and learning approaches as presage factors predicting performance directly and indirectly via the perceived educational environment.

Model fit was assessed using the root mean square error of approximation (RMSEA), comparative fit index (CFI), and Tucker-Lewis index (TLI). As recommended (Kline 2015), RMSEA < 0.06, CFI > 0.95, and TLI > 0.95 were taken to indicate acceptable model fit. Akaike’s information criterion (AIC) was used to determine which model fitted the data best, with lower values indicating better fit.

Indirect (mediated) effects were tested using bootstrapping with 2000 iterations, and 95% confidence intervals were calculated. The variables that were used in the interaction term (conscientiousness and the perceived environment) were mean centered before multiplication for ease of interpretation. Interaction effects were probed using simple slope analysis. Each model included age, gender, parental education level, and prior performance as covariates.

Direct and indirect effects were interpreted in terms of the magnitude of the standardized beta coefficients, using the benchmarks proposed by (Keith 2014): > 0.05 is a small but meaningful direct effect, > 0.10 is a moderate direct effect, and > 0.25 is a large direct effect. As an indirect effect is the product of two effects (Kenny 2021), 0.003 (0.05 × 0.05) can be considered as a small but meaningful indirect effect, 0.01 (0.10 × 0.10) as a moderate indirect effect, and 0.06 (0.25 × 0.25) as a large indirect effect.

All analyses were carried out using SPSS and Amos Version 25 for Windows (SPSS Inc., Chicago, IL).

## Results

### Preliminary analyses

Data were obtained from 268 individuals. In this sample, the internal consistency of all questionnaire measures was high (Cronbach’s alphas = 0.86, 0.91, 0.79, and 0.75 for conscientiousness, DREEM, and deep and surface learning approaches, respectively). There were some missing data for 15 individuals (≤ 15 cases without performance data; 15 cases without parental education information). Little’s test applied to these variables was not significant (*χ*2 = 3.44, *df* = 2, *p* = 0.179), suggesting the data were missing completely at random. Missing data were therefore imputed using the maximum likelihood estimation method.

Half of the sample were repeaters, and around three-quarters of the sample had at least one parent educated to university level. As shown in Table 1, both conscientiousness and the perceived educational environment were associated with students’ deep and surface learning approaches, and performance on the CBA (all *p* values < 0.01). Repeater status, prior performance, and a deep learning approach were also associated with performance on the CBA (all *p* values < 0.01). Only gender, prior performance, and CBA performance were associated with performance on the OSCE (all *p* values < 0.01).

**Table 1:** Descriptive statistics and correlations for variables included in the models (N = 268)

	*M* (*SD*)	1.	2.	3.	4.	5.	6.	7.	8.	9.	10.
1. Age	20.80 (1.88)										
2. Gender, % (n) male	40.30 (108)	− 0.02									
3. Parental education, % (n) university	76.87 (206)	0.01	− 0.01								
4. Repeater status, % (n) repeater	50.37 (135)	0.19*	0.08	− 0.05							
5. Prior performance	71.49 (8.10)	0.02	− 0.06	0.07	− 0.30**						
6. Conscientiousness	33.27 (7.00)	0.02	0.08	− 0.07	0.03	0.16**					
7. Deep learning approach	32.56 (6.25)	0.13*	0.02	− 0.08	− 0.00	0.12	0.45**				
8. Surface learning approach	23.56 (5.81)	− 0.05	− 0.02	− 0.02	0.05	− 0.03	− 0.24**	− 0.38**			
9. Perceived educational environment	133.29 (17.90)	− 0.04	− 0.07	0.05	− 0.20**	0.19**	0.29**	0.38**	− 0.38**		
10. CBA performance	73.49 (8.99)	0.01	− 0.00	− 0.02	− 0.22**	0.62**	0.32**	0.25**	− 0.07	0.18**	
11. OSCE performance	79.44 (7.20)	− 0.06	0.32**	− 0.06	− 0.12*	0.29**	0.02	− 0.06	0.00	0.04	0.25**

CBA computer-based assessment, OSCE Objective Structured Clinical Examination

Values are means (standard deviations), unless stated otherwise

*p<0.05, **p<0.01

All correlations between variables to be included as presage factors in the models were small to moderate (*r* range 0.13–0.62), indicating that multicollinearity was not an issue. All variables to be included in the models were normally distributed (skewness < 1, kurtosis ≤ 2).

### Path analysis

Model 1 had acceptable fit: *χ*^2^ (2) = 0.23, *p* = 0.89, CFI = 1.00, TLI = 1.11, RMSEA = 0.00. Model 2 (with the added interaction term) also fitted the data: *χ*^2^ (2) = 0.23, *p* = 0.89, CFI = 1.00, TLI = 1.12, RMSEA = 0.00. However, model 1 had a lower AIC value than model 2 (150.23 vs. 176.23), suggesting better fit. Model 3 did not fit the data: *χ*^2^ (1) = 4.49, *p* = 0.03, CFI = 0.99, TLI = 0.65, RMSEA = 0.11. Table 2 provides the standardized beta (β) coefficients for direct and indirect associations among presage, process, and product variables in model 1.

**Table 2:** Standardized beta coefficients for model 1

Presage factors	Process factors		Product factors					
	DA	SA	CBA			OSCE		
	Direct effect	Direct effect	Direct effect	Indirect effect via DA	Indirect effect via SA	Direct effect	Indirect effect via DA	Indirect effect via SA
Age	0.14*	− 0.05	− 0.01	0.18*	− 0.02	− 0.02	− 0.15	0.01
Gender	0.02	− 0.03	0.02	0.04	− 0.02	0.35***	− 0.03	0.01
Parental education	− 0.06	− 0.02	− 0.04	− 0.16	− 0.02	− 0.10	0.13	0.01
Prior performance	0.02	0.05	0.57***	0.00	0.00	0.32***	− 0.00	− 0.00
Conscientiousness	0.35***	− 0.14*	0.19***	0.05*	− 0.01	− 0.04	− 0.04	0.00
Perceived educational environment	0.30***	− 0.36***	− 0.02	0.02*	− 0.01	0.06	− 0.02	0.00

*DA* deep learning approach, *SA* surface learning approach, *CBA* computer-based assessment, *OSCE* objective structured clinical examination

*p < 0.05, **p < 0.01, ***p < 0.001

#### Model 1

**Fig. 1.**
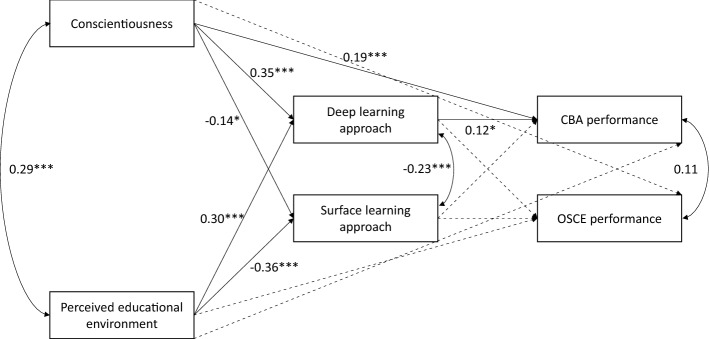
Paths tested within Model 1. Standardized coefficients are included for statistically significant paths. Dashed lines indicate non-significant paths. Covariates are not included for readability. *p < 0.05; **p < 0.01; ***p < 0.001. N = 268

Figure 1 depicts model 1, along with significant paths. In model 1, the presage variables accounted for 29% of variance in deep learning approach, and 17% of variance in surface learning approach. The presage and process variables accounted for 45% of variance in CBA performance, and 23% of variance in OSCE performance.

In terms of direct effects, conscientiousness and the perceived educational environment positively predicted a deep learning approach, with large effect sizes (β = 0.35 and 0.30, respectively); and negatively predicted a surface learning approach, with a moderate effect size for conscientiousness (β = −0.14) and a large effect size for the perceived educational environment (β = − 0.36). A deep learning approach moderately predicted CBA performance (β = 0.12), but not OSCE performance. Conscientiousness, but not the perceived educational environment, directly predicted CBA performance, with a moderate effect size (β = 0.19). Neither conscientiousness nor the perceived educational environment directly predicted OSCE performance. Performance in the previous semester directly and strongly predicted both CBA and OSCE performance (β = 0.57 and 0.32, respectively). In terms of covariates, age positively and moderately predicted a deep learning approach (β = 0.14). There was also a large effect of gender on OSCE performance (β = 0.35), with females performing better than males.

In terms of indirect effects, there was a moderate to large indirect path from conscientiousness through a deep learning approach to CBA performance (β = 0.05; 95% CI 0.01–0.11). There was also a moderate path from the perceived educational environment through a deep learning approach to CBA performance (β = 0.02; 95% CI 0.00–0.04).

#### Model 2

**Fig. 2.**
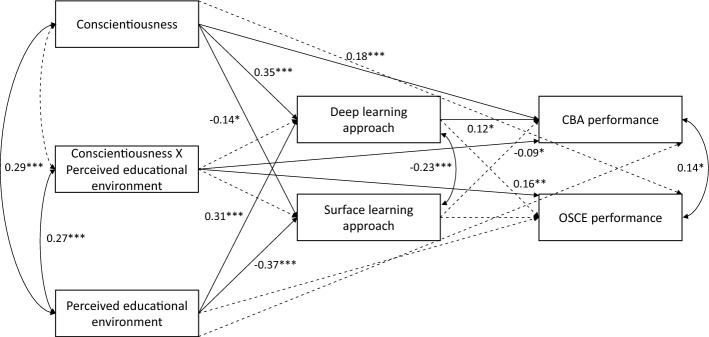
Paths tested within Model 2. Standardized coefficients are included for statistically significant paths. Dashed lines indicate non-significant paths. Covariates are not included for readability. *p < 0.05; **p < 0.01; ***p < 0.001. N = 268

**Fig. 3.**
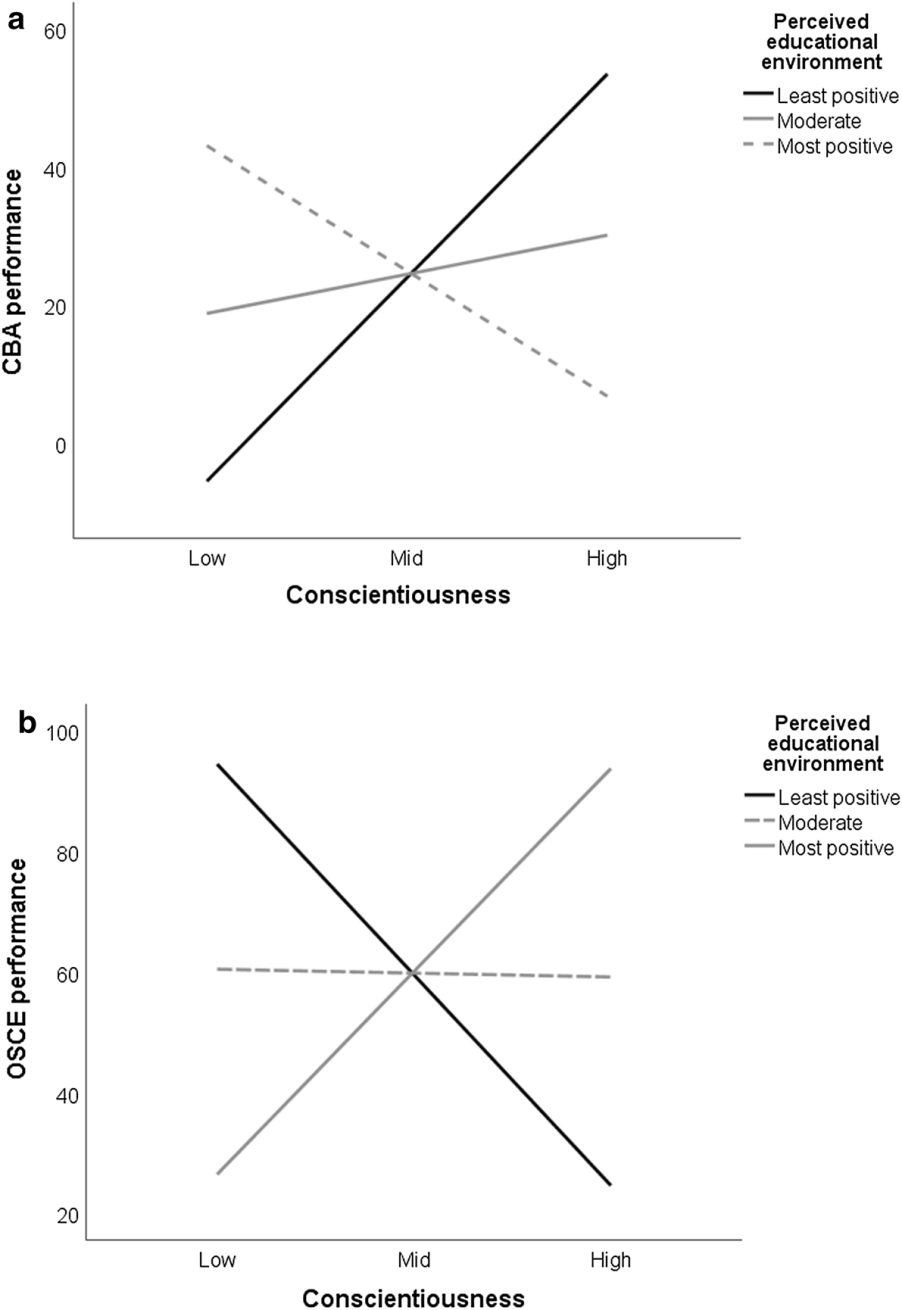
Mean CBA and OSCE performance at each level of conscientiousness (low = 1*SD* below the mean, moderate = average, and high = 1*SD* above the mean) by each level of the perceived educational environment (least positive = 1*SD* below the mean, moderate = average, and most positive = 1*SD* above the mean). Bold lines indicate significant effects of conscientiousness on performance at the *p* < 0.05 level. Dashed lines indicate non-significant effects. *CBA* computer-based assessment, *OSCE* objective structured clinical examination

Figure 2 depicts model 2, along with significant paths. Figure 3 depicts the interaction effects. For model 2, the parameter estimates were almost identical to those for model 1. In addition to the effects outlined for model 1, there was a moderate direct interaction effect (conscientiousness X perceived educational environment) on CBA and OSCE performance (β = − 0.09, *p* = 0.05; β = 0.16, *p* < 0.01, respectively). For CBA performance, there was a positive association between conscientiousness and performance when perceptions of the educational environment were the least positive (β = 1.21; 95% CI 0.14 – 2.37, *p* < 0.05) or moderate (β = 0.23; 95% CI 0.10–0.36, *p* < 0.01), but there was no significant association when perceptions of the educational environment were the most positive (β = − 0.75; 95% CI − 1.89 to 0.31, *p* = 0.17). For OSCE performance, there was a positive association between conscientiousness and performance when perceptions of the educational environment were the most positive (β = 1.38; 95% CI 0.29–2.41, *p* < 0.05), a negative association when perceptions of the educational environment were the least positive (β = − 1.43; 95% CI − 2.55 to − 0.33, *p* < 0.05), and no significant association when perceptions were moderate (β = − 0.03; 95% CI − 0.17 to 0.11, *p* = 0.68). The interaction term accounted for an additional 1% in the statistical prediction of CBA performance, and an additional 2% in the statistical prediction of OSCE performance.

## Discussion

This is the first study to examine conscientiousness and the perceived educational environment as independent and interactive predictors of performance in medical school. Model 1, which tested the main pathways in the 3P model (Biggs, 1993a), provided the best fit to the data. Both conscientiousness and the perceived educational environment predicted performance on the computer-based assessment (CBA) at the end of the third year, via a deep learning approach. Neither conscientiousness nor the perceived educational environment independently predicted performance on the Objective Structured Clinical Examination (OSCE). Model 2, with the added interaction terms, had acceptable but less optimal fit to the data compared with model 1. In this model, there was a cross-over interaction effect: conscientiousness positively predicted OSCE performance among students with the most positive perceptions of the educational environment, but negatively predicted performance among those with the least positive perceptions of the educational environment. There was also a significant interaction effect for CBA performance, but in a different direction: conscientiousness positively predicted performance among those with the least positive or moderate perceptions of the educational environment, but there was no effect of conscientiousness among those with the most positive perceptions. Taken together, these findings highlight the importance of considering both student characteristics and perceptions of the educational context when investigating predictors of performance.

Consistent with the main pathways proposed in the 3P model (Biggs, 1993a), conscientiousness (an intra-personal presage factor), and the perceived educational environment (a contextual presage factor) predicted CBA performance directly, and/or indirectly via a deep approach to learning (a process factor). These findings corroborate previous research examining high-school students’ performance within the 3P framework (Ginns et al., 2014, 2018), and provide further support that a deep approach to learning positively predicts medical students’ performance on CBAs (Reid et al., 2007). However, there were no effects of approaches to learning (direct or indirect) on OSCE performance. Some previous studies have found an association between a deep learning approach and clinical exam performance (May et al., 2012; McManus et al., 1998), although several other studies found no association (Arnold & Feighny, 1995; Lohuizen et al., 2009; Martin et al., 2000). A deep learning approach, which has an emphasis on thorough understanding, may be most suited to knowledge acquisition (which is assessed in the CBA). However, it may be less useful for demonstrating clinical and communication skills, which are assessed in the OSCE. Research indicates that students tend to use more than one approach to learning (Lohuizen et al., 2009), and vary their approach according to the assessment context (Newble & Jaeger 1983; Thomas & Bain 1984; Piumatti et al., 2021). In a clinical assessment context, successful students may be more likely to use a combination of strategies that support the integration of knowledge, skills, and professional behavior. Some research indicates that a strategic approach to learning, which was not assessed in the present study, positively predicts OSCE performance (Martins et al., 2000).

In addition to the main predictive effects (presage to process to product), the 3P model proposes that there are reciprocal relationships between presage, process, and product factors, which operate over time: process variables can predict some presage variables, and outcomes can predict future process and presage variables. In the present study, model 3 (which identified learning approaches and conscientiousness as predictors of the perceived educational environment) did not adequately fit the data. In model 1, performance in the previous semester was strongly associated with a deep learning approach and both CBA and OSCE performance at the end of the third year. However, longitudinal studies are needed to provide a robust test of reciprocal relationships between presage and product factors (Fryer & Ginns, 2018).

The findings of this study extend previous research by examining interactive effects of personality and the perceived educational environment on medical student performance. No known studies have examined how the perceived educational environment might moderate the effects of personality on performance, therefore it is not possible to make direct comparisons. However, some previous work has shown that the effects of conscientiousness on performance vary according to the medical school context, with conscientiousness positively predicting pre-clinical, but not clinical knowledge (Ferguson et al., 2003, 2014). The present findings suggest that both conscientiousness and perceptions of the educational environment need to be high to enhance OSCE performance; but conscientiousness is sufficient to enhance CBA performance even among those who have lower perceptions of the educational environment. Perhaps this can be partly explained by the different demands of the CBA and OSCE: conscientiousness can enhance performance when the task is suited to learning facts and a methodological approach (as in the CBA), but is insufficient when the task is more complex, requiring knowledge application and communication skills (such as in a clinical assessment context). Taken together, these findings are in line with Trait Activation Theory, according to which the behavioral expression of traits depends on the presence of trait-relevant contextual cues (Tett et al, 2013; 2021). However, it is important to note that the interaction effects explained a small proportion of variance in CBA and OSCE performance; therefore, it will be important further examine these effects in a larger sample.

Although the findings of the present study require replication, at face value they highlight the need for strategies to enhance both conscientiousness and perceptions of the educational environment. Selecting students who score highly on conscientiousness upon entry to medical school is limited given that traits change over time and their expression is context-dependent (Ferguson & Lievens, 2017). Some research indicates that performing trait-typical behaviors predicts trait change over time (Hudson et al., 2019; Hudson & Fraley, 2015); however, no research has examined whether personality management could be included as part of medical education. Stress-management interventions, and/or those that enhance students’ self-efficacy may improve performance (Shapiro et al., 2000; Stegers‐Jager et al., 2012). A student-centered learning environment should take perceived contextual factors into account by providing support, from both an academic and social perspective. Setting clear goals, highlighting the value of educational activities, encouraging participation, providing possibilities for independent study, and maintaining an appropriate workload and information load are factors that can enhance motivation and performance (Baeten et al., 2010; Pelaccia and Viau 2017). Students can be supported socially through learning communities, which may help them find more connections through medical school (Ferguson et al., 2009). Research has shown that perceptions of the educational environment are more positive at medical schools with learning communities than at those without (Smith et al., 2016). Providing adequate support during the transition from pre-clinical to clinical studies may be particularly important for students’ academic performance and well-being. Some research indicates that perceptions of the educational environment decline the most during the third year, as students transition to the clinical phase of medical training (Dunham et al., 2017).

### Limitations and suggestions for future research

This study focused on the personality trait conscientiousness, which has been the most consistently linked to medical student performance in the literature (Doherty & Nugent 2011; Lievens et al., 2002, 2009). Nevertheless, other traits such as extraversion and openness have been linked to performance, especially during the clinical years of medical school (Lievens et al., 2009; Sobowale et al., 2018). These traits should be considered in future research testing the models in this study, with larger sample sizes. This study also focused on the perceived educational environment, which is an important but subjective predictor of student performance. It will be important to examine how objective features of the educational environment fit within the tested models. Recent research found that a problem-based-learning environment and an integrated curriculum at medical school were associated with deeper approaches to learning (compared to a lecture-based environment, and a traditional curriculum), and this was mediated by student perceptions of the educational environment (Gustin et al., 2018). Motivational factors should also be considered as potential mediators and moderators of the pathways tested in this study. Student motivation is a key predictor of performance (Kusurkar et al., 2013; Wu et al., 2020), may explain the effect of conscientiousness and/or the perceived educational environment on performance (Pelaccia & Viau 2017; Richardson & Abraham 2009), and may compensate for low conscientiousness and/or less positive perceptions of the educational environment (Cheng & Ickes 2009). Self-report measures may be prone to bias; although research suggests that bias is more prevalent upon entry to medical school, when the stakes are high (Anglim et al., 2018). Finally, the cross-sectional nature of the analyses precludes inferences of causality, therefore future studies should longitudinally examine the models in this study.

### Conclusion

This study examined the independent and interactive effects of conscientiousness and the perceived educational environment in relation to medical students’ performance at the end of the third year. Taken together, the findings highlight the importance of jointly considering student traits and context-related factors when supporting medical students to enhance their performance. The identification of interactive effects is a novel finding that requires further investigation.

## References

[CR1] Anderson J, Gerbing D (1988). Structural equation modeling in practice: A review and recommended two-step approach. Psychological Bulletin.

[CR2] Anglim J, Bozic S, Little J, Lievens F (2018). Response distortion on personality tests in applicants: Comparing high-stakes to low-stakes medical settings. Advances in Health Sciences Education.

[CR3] Arnold L, Feighny KM (1995). Students’ general learning approaches and performances in medical school: A longitudinal study. Academic Medicine.

[CR4] Baeten M, Kyndt E, Struyven K, Dochy F (2010). Using student-centred learning environments to stimulate deep approaches to learning: Factors encouraging or discouraging their effectiveness. Educational Research Review.

[CR5] Biggs JB (1987). Student approaches to learning and studying. Research monograph.

[CR6] Biggs JB (1993). From theory to practice: A cognitive systems approach. Higher Education Research & Development.

[CR7] Biggs J (1993). What do inventories of students' learning processes really measure? A theoretical review and clarification. British Journal of Educational Psychology.

[CR8] Biggs J, Kember D, Leung DYP (2001). The revised two-factor Study Process Questionnaire: R-SPQ-2F. British Journal of Educational Psychology.

[CR9] Chamorro-Premuzic T, Furnham A (2008). Personality, intelligence and approaches to learning as predictors of academic performance. Personality and Individual Differences.

[CR10] Cheng W, Ickes W (2009). Conscientiousness and self-motivation as mutually compensatory predictors of university-level GPA. Personality and Individual Differences.

[CR11] Colbert-Getz JM, Kim S, Goode VH, Shochet RB, Wright SM (2014). Assessing medical students’ and residents’ perceptions of the learning environment: Exploring validity evidence for the interpretation of scores from existing tools. Academic Medicine.

[CR12] Costa P, McCrae R (1992). Normal personality assessment in clinical practice: The NEO personality inventory. Psychological Assessment.

[CR13] Dingemanse NJ, Kazem AJN, Réale D, Wright J (2010). Behavioural reaction norms: Animal personality meets individual plasticity. Trends in Ecology & Evolution.

[CR14] Diseth Å (2003). Personality and approaches to learning as predictors of academic achievement. European Journal of Personality.

[CR15] Doherty EM, Nugent E (2011). Personality factors and medical training: A review of the literature. Medical Education.

[CR16] Duff A, Boyle E, Dunleavy K, Ferguson J (2004). The relationship between personality, approach to learning and academic performance. Personality and Individual Differences.

[CR17] Dunham L, Dekhtyar M, Gruener G, CichoskiKelly E, Deitz J, Elliott D (2017). Medical student perceptions of the learning environment in medical school change as students transition to clinical training in undergraduate medical school. Teaching and Learning in Medicine.

[CR18] Entwistle N (2009). Teaching for understanding at university: Deep approaches and distinctive ways of thinking.

[CR19] Ferguson E, Heckman JJ, Corr P (2011). Personality and economics: Overview and proposed framework. Personality and Individual Differences.

[CR20] Ferguson E, James D, Madeley L (2002). Factors associated with success in medical school: Systematic review of the literature. British Medical Journal.

[CR21] Ferguson E, James D, O’Hehir F, Sanders A, McManus IC (2003). Pilot study of the roles of personality, references, and personal statements in relation to performance over the five years of a medical degree. British Medical Journal.

[CR22] Ferguson E, Lievens F (2017). Future directions in personality, occupational and medical selection: Myths, misunderstandings, measurement, and suggestions. Advances in Health Sciences Education.

[CR23] Ferguson E, Semper H, Yates J, Fitzgerald JE, Skatova A, James D (2014). The ‘dark side’ and ‘bright side’ of personality: When too much conscientiousness and too little anxiety are detrimental with respect to the acquisition of medical knowledge and skill. PLoS ONE.

[CR24] Ferguson KJ, Wolter EM, Yarbrough DB, Carline JD, Krupat E (2009). Defining and describing medical learning communities: Results of a national survey. Academic Medicine.

[CR25] Fryer LK, Ginns P (2018). A reciprocal test of perceptions of teaching quality and approaches to learning: A longitudinal examination of teaching-learning connections. Educational Psychology.

[CR26] Ginns P, Martin AJ, Papworth B (2014). Student learning theory goes (back) to (high) school. Instructional Science.

[CR27] Ginns P, Martin AJ, Papworth B (2018). Student learning in Australian high schools: Contrasting personological and contextual variables in a longitudinal structural model. Learning and Individual Differences.

[CR28] Gruppen LD, Stansfield RB (2016). Individual and institutional components of the medical school educational environment. Academic Medicine.

[CR29] Gustin M-P, Abbiati M, Bonvin R, Gerbase MW, Baroffio A (2018). Integrated problem-based learning versus lectures: A path analysis modelling of the relationships between educational context and learning approaches. Medical Education Online.

[CR30] Hudson NW, Briley DA, Chopik WJ, Derringer J (2019). You have to follow through: Attaining behavioral change goals predicts volitional personality change. Journal of Personality and Social Psychology.

[CR31] Hudson NW, Fraley RC (2015). Volitional personality trait change: Can people choose to change their personality traits?. Journal of Personality and Social Psychology.

[CR32] Karagiannopoulou E, Milienos FS (2015). Testing two path models to explore relationships between students’ experiences of the teaching–learning environment, approaches to learning and academic achievement. Educational Psychology.

[CR33] Keith TZ (2014). Multiple regression and beyond: An introduction to multiple regression and structural equation modeling.

[CR34] Kenny, D. A. (2021). Mediation. http://davidakenny.net/cm/mediate.htm#IE. Retrieved 5 October 2020

[CR35] Kline RB (2015). Principles and practice of structural equation modeling.

[CR36] Komarraju M, Karau SJ, Schmeck RR, Avdic A (2011). The Big Five personality traits, learning styles, and academic achievement. Personality and Individual Differences.

[CR37] Kusurkar RA, Ten Cate TJ, Vos CMP, Westers P, Croiset G (2013). How motivation affects academic performance: A structural equation modelling analysis. Advances in Health Sciences Education.

[CR38] Lee M, Vermillion M (2018). Comparative values of medical school assessments in the prediction of internship performance. Medical Teacher.

[CR39] Lievens F, Coetsier P, Fruyt FD, Maeseneer JD (2002). Medical students’ personality characteristics and academic performance: A five-factor model perspective. Medical Education.

[CR40] Lievens F, Ones DS, Dilchert S (2009). Personality scale validities increase throughout medical school. The Journal of Applied Psychology.

[CR41] Martin IG, Stark P, Jolly B (2000). Benefiting from clinical experience: the influence of learning style and clinical experience on performance in an undergraduate objective structured clinical examination. Medical Education.

[CR42] May W, Chung EK, Elliott D, Fisher D (2012). The relationship between medical students’ learning approaches and performance on a summative high-stakes clinical performance examination. Medical Teacher.

[CR43] McManus IC, Richards P, Winder BC, Sproston KA (1998). Clinical experience, performance in final examinations, and learning style in medical students: Prospective study. British Medical Journal.

[CR44] Muller FH, Louw J (2004). Learning environment, motivation and interest: Perspectives on self-determination theory. South African Journal of Psychology.

[CR45] Newble DI, Entwistle NJ (1986). Learning styles and approaches: Implications for medical education. Medical Education.

[CR46] Newble DI, Jaeger K (1983). The effect of assessments and examinations on the learning of medical students. Medical Education.

[CR47] Nijhuis J, Segers M, Gijselaers W (2007). The interplay of perceptions of the learning environment, personality and learning strategies: A study amongst International Business Studies students. Studies in Higher Education.

[CR48] O’Neill LD, Wallstedt B, Eika B, Hartvigsen J (2011). Factors associated with dropout in medical education: A literature review. Medical Education.

[CR49] Papadakis MA, Teherani A, Banach MA, Knettler TR, Rattner SL, Stern DT (2005). Disciplinary action by medical boards and prior behavior in medical school. The New England Journal of Medicine.

[CR50] Pelaccia T, Viau R (2017). Motivation in medical education. Medical Teacher.

[CR51] Piumatti G, Abbiati M, Gerbase MW, Baroffio A (2021). Patterns of change in approaches to learning and their impact on academic performance among medical students: A longitudinal analysis. Teaching and Learning in Medicine.

[CR52] Platt JR (1964). Strong inference. Science.

[CR53] Price L, Gijbels D, Donche V, Richardson JTE, Vermunt JD (2014). Modelling factors for predicting student learning outcomes in higher education. Learning patterns in higher education: Dimensions and research perspectives.

[CR54] Price L, Richardson JTE, Rust C (2004). Why is it difficult to improve student learning?. Improving student learning: Theory, research and scholarship.

[CR55] Ramsden P (1991). A performance indicator of teaching quality in higher education: The Course Experience Questionnaire. Studies in Higher Education.

[CR56] Reid WA, Duvall E, Evans P (2007). Relationship between assessment results and approaches to learning and studying in year two medical students. Medical Education.

[CR57] Richardson JTE (2006). Investigating the relationship between variations in students’ perceptions of their academic environment and variations in study behaviour in distance education. British Journal of Educational Psychology.

[CR58] Richardson M, Abraham C (2009). Conscientiousness and achievement motivation predict performance. European Journal of Personality.

[CR59] Richardson M, Abraham C, Bond R (2012). Psychological correlates of university students’ academic performance: A systematic review and meta-analysis. Psychological Bulletin.

[CR60] Roff S, McAleer S, Harden RM, Al-Qahtani M, Ahmed AU, Deza H (1997). Development and validation of the Dundee Ready Education Environment Measure (DREEM). Medical Teacher.

[CR61] Rolland JP, Parker WD, Stumpf H (1998). A psychometric examination of the French translations of NEO-PI-R and NEO-FFI. Journal of Personality Assessment.

[CR62] Rubright JD, Jodoin M, Barone MA (2019). Examining demographics, prior academic performance, and United States Medical Licensing Examination Scores. Academic Medicine.

[CR63] Sadlo G, Richardson JTE (2003). Approaches to studying and perceptions of the academic environment in students following problem-based and subject-based curricula. Higher Education Research and Development.

[CR64] Shapiro SL, Shapiro DE, Schwartz GE (2000). Stress management in medical education: A review of the literature, 1969 to 1998. Academic medicine.

[CR65] Smith SD, Dunham L, Dekhtyar M, Dinh A, Lanken PN, Moynahan KF (2016). Medical student perceptions of the learning environment: Learning communities are associated with a more positive learning environment in a multi-institutional medical school study. Academic Medicine.

[CR66] Sobowale K, Ham SA, Curlin FA, Yoon JD (2018). Personality traits are associated with academic achievement in medical school: A nationally representative study. Academic Psychiatry.

[CR67] Soemantri D, Herrera C, Riquelme A (2010). Measuring the educational environment in health professions studies: A systematic review. Medical Teacher.

[CR68] Stegers-Jager KM, Cohen-Schotanus J, Themmen APN (2012). Motivation, learning strategies, participation and medical school performance. Medical Education.

[CR69] Stegers-Jager KM, Themmen APN, Cohen-Schotanus J, Steyerberg EW (2015). Predicting performance: Relative importance of students’ background and past performance. Medical Education.

[CR70] Tett, R. P., Simonet, D. V., Walser, B., & Brown, C. (2013). Trait activation theory. *Handbook of personality at work*, 71–100.

[CR71] Tett RP, Toich MJ, Ozkum SB (2021). Trait Activation Theory: A review of the literature and applications to five lines of personality dynamics research. Annual Review of Organizational Psychology and Organizational Behavior.

[CR72] Thomas P, Bain J (1984). Contextual dependence of learning approaches. Human Learning.

[CR73] van Lohuizen MT, Kuks JBM, van Hell EA, Raat AN, Cohen-Schotanus J (2009). Learning strategies during clerkships and their effects on clinical performance. Medical Teacher.

[CR74] Wayne SJ, Fortner SA, Kitzes JA, Timm C, Kalishman S (2013). Cause or effect? The relationship between student perception of the medical school learning environment and academic performance on USMLE Step 1. Medical Teacher.

[CR75] Wu H, Li S, Zheng J, Guo J (2020). Medical students’ motivation and academic performance: the mediating roles of self-efficacy and learning engagement. Medical Education Online.

[CR76] Yates, J., & James, D. (2010). Risk factors at medical school for subsequent professional misconduct: Multicentre retrospective case-control study. *British Medical Journal*, *340*, c2040. 10.1136/bmj.c2040 PMC3191727 20423965

